# Barcoding of Chrysomelidae of Euro-Mediterranean area: efficiency and problematic species

**DOI:** 10.1038/s41598-018-31545-9

**Published:** 2018-09-07

**Authors:** Giulia Magoga, Didem Coral Sahin, Diego Fontaneto, Matteo Montagna

**Affiliations:** 1 Dipartimento di Scienze Agrarie e Ambientali - Università degli Studi di Milano, Via Celoria 2, 20133 Milano, Italy; 2 Directorate of Plant Protection Central Research Institute, Yenimahalle, Ankara Turkey; 3 Consiglio Nazionale delle Ricerche-Istituto per lo Studio degli Ecosistemi, Largo Tonolli 50, 28922 Verbania, Italy

## Abstract

Leaf beetles (Coleoptera: Chrysomelidae), with more than 37,000 species worldwide and about 2,300 in the Euro-Mediterranean region, are an ecological and economical relevant family, making their molecular identification of interest also in agriculture. This study, part of the Mediterranean Chrysomelidae Barcoding project (www.c-bar.org), aims to: (*i*) develop a reference Cytochrome c oxidase I (COI) library for the molecular identification of the Euro-Mediterranean Chrysomelidae; (*ii*) test the efficiency of DNA barcoding for leaf beetles identification; (*iii*) develop and compare optimal thresholds for distance-based identifications estimated at family and subfamily level, minimizing false positives and false negatives. Within this study, 889 COI nucleotide sequences of 261 species were provided; after the inclusion of information from other sources, a dataset of 7,237 sequences (542 species) was analysed. The average intra-interspecific distances were in the range of those recorded for Coleoptera: 1.6–24%. The estimated barcoding efficiency (~94%) confirmed the usefulness of this tool for Chrysomelidae identification. The few cases of failure were recorded for closely related species (e.g., *Cryptocephalus marginellus* superspecies, *Cryptocephalus violaceus* - *Cryptocephalus duplicatus* and some *Altica* species), even with morphologically different species sharing the same COI haplotype. Different optimal thresholds were achieved for the tested taxonomic levels, confirming that group-specific thresholds significantly improve molecular identifications.

## Introduction

Chrysomelidae, or leaf beetles, is one of the most species-rich families of Coleoptera. Leaf beetles are distributed worldwide (except Antarctica) and inhabit almost all habitats presenting vegetation. Leaf beetles include more than 37,000 species at global level belonging to more than 2,000 genera^1^. In the Palearctic region approximately 3,500 species have been described so far^2^, and about 2,300 of them occur in the Euro-Mediterranean region^3–5^. With few exceptions, leaf beetles are phytophagous insects adapted to feed on plant species, including some of agricultural interest (e.g., *Diabrotica virgifera* LeConte, 1868 and *Leptinotarsa decemlineata* Say, 1824). The species-specific strict association with the host plants makes leaf beetles an interesting group for evolutionary studies (e.g.^6,7^); however, they have received attention also due to their impact on agriculture (e.g.^8^) and to their use as biological control agents of invasive plants^9,10^. The correct identification of organisms is regarded to be essential in both applicative field and evolutionary studies; at present, their taxonomy, based on morphological features, requires a high level of expertise that could be reached only after years of study. In some cases, as for several species groups, the accurate species identification of adults can only be achieved extracting genitalia^11–13^. Therefore, preimaginal developmental stages can be only rarely identified to species level. Thus, approaches based on morphology may not be efficient for beetles identification and become strongly time consuming especially in large scale studies, for example in biomonitoring surveys for agricultural biocontrol.

DNA based approaches have emerged as useful tools for the identification of organisms^14,15^, and the efficacy of the cytochrome c oxidase subunit I (COI) marker in molecular identification of Coleoptera (including leaf beatles) was demonstrated^16–18^. At present, on-line databases harbour about 8,000 COI sequences assigned to approximately 1,200 leaf beetles species worldwide, roughly about 4% of the overall described species. We are still far from having a reliable reference database, and most of the detailed barcoding studies for European leaf beetles have been developed within limited geographic contexts^19,20^. In order to increase the number of barcoded species, including also the rare ones, large scale biodiversity studies focused on leaf beetles inhabiting different biogeographic regions are needed. The Mediterranean Chrysomelidae Barcoding project (C-bar; www.c-bar.org; ^3^), started in 2009 and, involving many taxonomists and specialists of different subfamilies, aims to develop a reference database of sequences for the molecular identification of leaf beetles inhabiting the Euro-Mediterranean region. In the present study, we analysed the dataset of COI gene sequences obtained within the C-bar project with the purpose of: (i) evaluate the efficiency of the DNA barcoding dataset; (ii) estimate the optimal intraspecific and interspecific thresholds for the identification of leaf beetles species at different taxonomic level (i.e., family *vs* subfamily level).

## Materials and Methods

### Ethics Statement

No species of Coleoptera Chrysomelidae are listed in national laws as protected or endangered. All the specimens were collected between 2009–2013 in state-owned properties. The collection of these invertebrates is not subjected to restriction by national or international laws and does not require special permission. All the organisms were collected before the approval of Nagoya protocol 283/2014/UE.

### Sample collection and identification

Leaf beetles were collected in sampling campaigns occurred from 2009 to 2013 in different ecoregions of central and southern Europe and North Africa. The animals were collected using different methods: from the vegetation by sweep net or by beating sheet, and directly by hand in specific habitats. All the specimens were stored in absolute ethanol in order to preserve the genomic DNA and preserved at −20 °C. Specimen manipulation and dissection (when necessary) were completed with the auxiliary use of a stereomicroscope Leica MS5, a compound microscope Zeiss Axio Zoom V16, and images were acquired with the digital camera Zeiss Axiocam 506. The specimens were morphologically identified by the authors and other expert taxonomists. The nomenclature adopted in this study follows that of the European Fauna (https://fauna-eu.org/).

### DNA extraction, PCR and sequencing

DNA extractions were performed in two laboratories (the Biodiversity Institute of Ontario - University of Guelph; the Laboratory of Molecular Entomology at Dipartimento di Scienze Agrarie e Ambientali - Università degli Studi di Milano) adopting the following different protocols: (i) DNA extraction from one hind leg of the specimen, and (ii) DNA extraction from the whole body, in both cases using Qiagen DNeasy Blood and Tissue Kit (Qiagen, Hilden, Germany) as reported in Magoga *et al*.^3^.

After DNA extraction, the voucher specimens were dry mounted on pins in the case of whole body DNA extraction, or preserved in absolute ethanol at −30 °C in the case of DNA extraction from a single leg. An aliquot of the extracted DNA was preserved in both laboratories at −80 °C as reference. The standard barcode region of the mitochondrial COI was amplified by PCR using standard barcode primers LCO1490/HCO2198^21^. In case of unsuccessful amplifications, the alternative COI primers LepF1/LepR1 were adopted to amplify the selected region^22^. PCRs were performed in a volume of 25 μL reaction mix containing: 1X GoTaq reaction Buffer (10 mM Tris-HCl at pH 8.3, 50 mM KCl and 1.5 mM MgCl_2_), 0.2 mM of each deoxynucleoside triphosphate, 0.5 pmol of each primer, 0.3 U of GoTaq DNA Polymerase and 10/20 ng of template DNA. The adopted thermal protocol is reported in Montagna *et al*.^11^. Positive amplicons were directly sequenced on both strands using the marker-specific primers from ABI technology (Applied Biosystems, Foster City, USA). Consensus sequences were obtained editing electropherograms using Geneious R8 (Biomatters Ltd., Auckland, New Zealand. License owned by Matteo Montagna). Spurious amplifications of COI sequences were checked using Standard Nucleotide BLAST^23^. The presence of open reading frame was verified for the obtained sequences by using the on-line tool EMBOSS Transeq (http://www.ebi.ac.uk/Tools/st/emboss_transeq/), then sequences were aligned at codon level using MUSCLE^24^ in MEGA 6.06^25^. Consensus sequences were deposited in the Bold Systems^26^ and in the European Nucleotide Archive to make them available for future studies (accession numbers reported in Table S1).

### Sequence mining and dataset development

Accession numbers of orthologous sequences belonging to European and Mediterranean Leaf Beatles species were recovered from previously published DNA barcoding studies^19,20,27^ and used to download the corresponding nucleotide sequences from public repositories (i.e., BOLD and GeneBank); this operation was completed using the R 3.3.3 (R Core Team, 2017) library *ape* v4.1^28^ and *rentrez* v1.1.0^29^.

Overall a total of 6,348 COI gene sequences were retrieved from public repositories. These nucleotide sequences and those obtained in the present study were organised in two datasets: (*i*) dataset *DS1*, composed only by the nucleotide sequences developed in this study; (*ii*) dataset *DS2*, composed by the sequences mined from online databases plus dataset *DS1*. We keep separated the two datasets in order to evaluate the efficiency of the here developed dataset, and to estimate the barcoding efficiency for the whole family using available COI sequences (*DS2*).

Taxonomy was standardised checking for the presence of synonymous names and assigning only one name (the accepted one following European Fauna https://fauna-eu.org/ nomenclature) to each species. *DS1* and *DS2* were also split in sub-datasets in order to obtain datasets including only one leaf beetles subfamily each. Only subfamily level datasets consisting of at least 2 species were retained. The procedure led to obtain datasets for the following ten subfamilies: Alticinae, Cassidinae, Chrysomelinae, Criocerinae, Cryptocephalinae, Donaciinae, Eumolpinae, Galerucinae, Hispinae and Orsodacninae.

### Bioinformatic analyses

For all the morphologically identified species of *DS1* and *DS2*, intraspecific and interspecific nucleotide divergences were calculated starting from a pairwise distance matrix developed using R library *spider* v1.1-5^30^ adopting Kimura-two parameters (K2P) as substitution model^31^. With the same R package a Threshold optimisation analysis was performed on *DS1*, *DS2* and on each subfamily-level dataset in order to calculate the value of nucleotide distance (optimal threshold; OT) that minimises the error related to molecular identification. This error is caused by the discordance between morphological and molecular identification and is called cumulative error (CE), calculated as the sum of the number of false positives (FP, conspecifics with a value of nucleotide divergence higher than the threshold value) plus the number of false negatives (FN, heterospecifics with a value of nucleotide divergence lower than the threshold value)^32^. Differences in CE values estimated at family and subfamily level were assessed using Student *t* test. The efficiency of molecular identification was estimated performing Best Close Match analyses, defined by Meier *et al*.^33^, on *DS1* and *DS2* (family level). The method compares each sequence of dataset with the others included in it and checks if the best matches (i.e., pairs of sequences with the lowest values of nucleotide distance) are between sequences of organisms morphologically identified as the same species. Each best match results in one of the following four states: “correct”, when the two closest sequences under the defined threshold belong to the same species; “incorrect”, the opposite situation; “ambiguous”, when the closest match is represented by more than one species; and, “no id” when no match is recorded under the chosen threshold.

For some groups of closely related species, where several misidentifications were observed, minimum-spanning haplotype networks^34^ were reconstructed using PopArt^35^.

## Results

### General features

**Figure 1:**
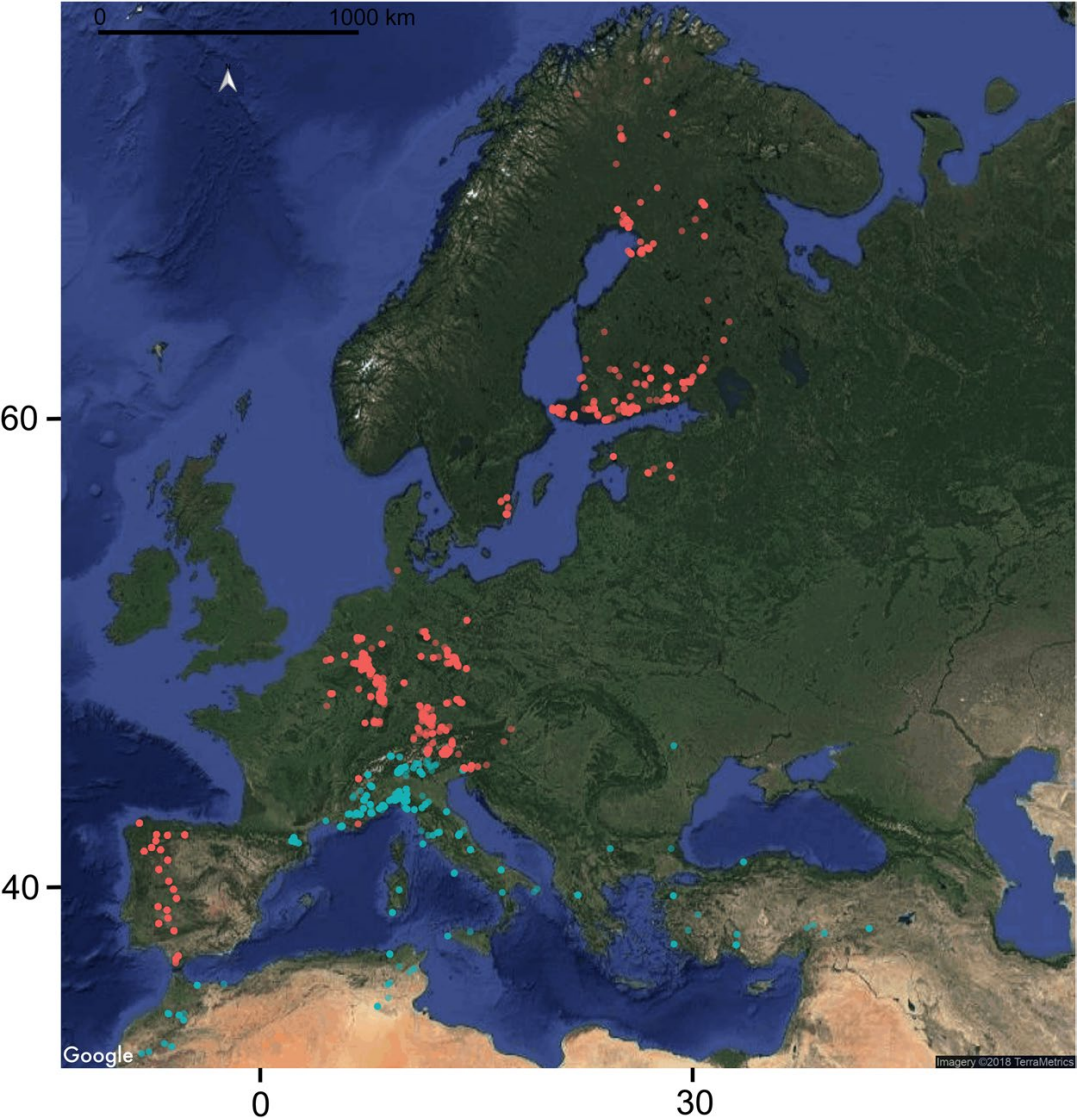
Collection sites of the individuals analysed in this study. Sampling localities of the individuals processed in this study (light blue dots) and whose barcodes were mined from online databases (orange dots). Map developed using R libraries *ggmap*^65^, *ggplot2*^66^, *ggsn*^67^; background image downloaded using the cited libraries from Google Imagery©2018 TerraMetrics.

The dataset developed in this study (i.e., *DS1*) consists of 889 COI sequences (average 654 bp [range: 494–658]), with a base composition of A = 29.4%; C = 19.6%; G = 16.1%; T = 34.9%. The dataset includes sequences of 261 leaf beetles species, the 11.4% of the Euro-Mediterranean species (74 singletons), belonging to 64 genera collected from ten countries within the Euro-Mediterranean region (Fig. 1 and Table S1). Out of the 261 barcoded species, COI sequences of 52 species were not already present in any online repository (Table S4).

Dataset *DS2*, consisting of the previously available sequences with the addition of *DS1*, is composed by 7,237 COI sequences (average 652 bp [range: 460–658]); with a base composition of A = 29.6%; C = 18.7%; G = 16.1%; T = 35.5%. In *DS2* the COI sequences of 542 species (~24% of the Euro-Mediterranean fauna) sampled in 19 different countries of Europe and North Africa are included (Fig. 1 and Table S5).

### Morphospecies intra-interspecific nucleotide distance

**Figure 2:**
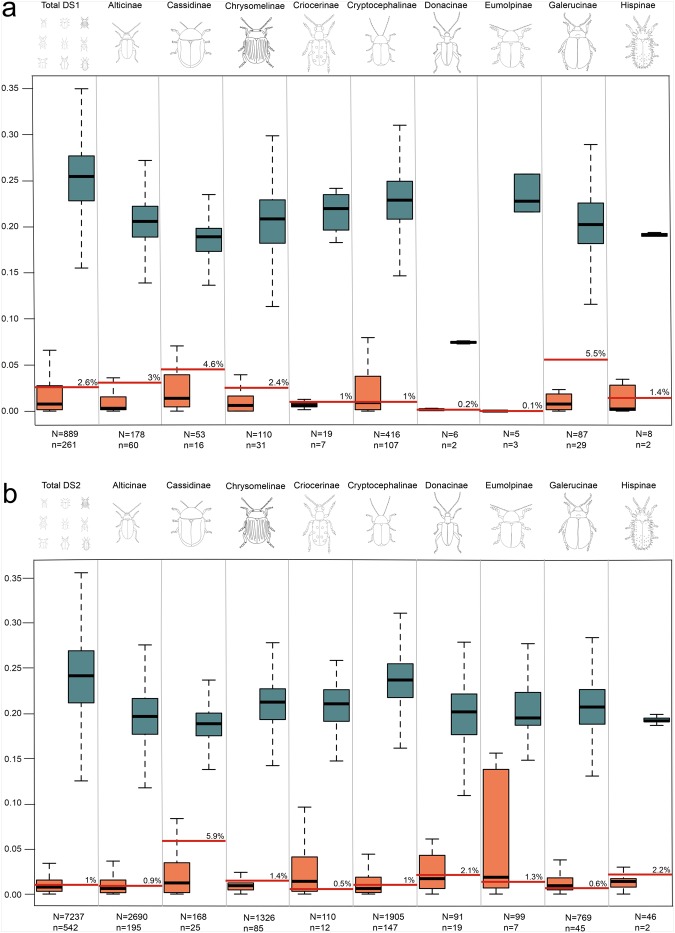
Boxplots of K2P inter-intraspecific pairwise nucleotide distances inferred from *DS1* (**a**) and *DS2* (**b**) datasets. Estimated intraspecific (orange) and interspecific (cadet blue) nucleotide distances are reported for each dataset at family and subfamily levels; optimal thresholds are reported as percentage and indicated by the red horizontal lines; below each bar the number of sequences (N) and of species (n) are reported. Above the bars datasets identifiers are reported.

**Figure 3:**
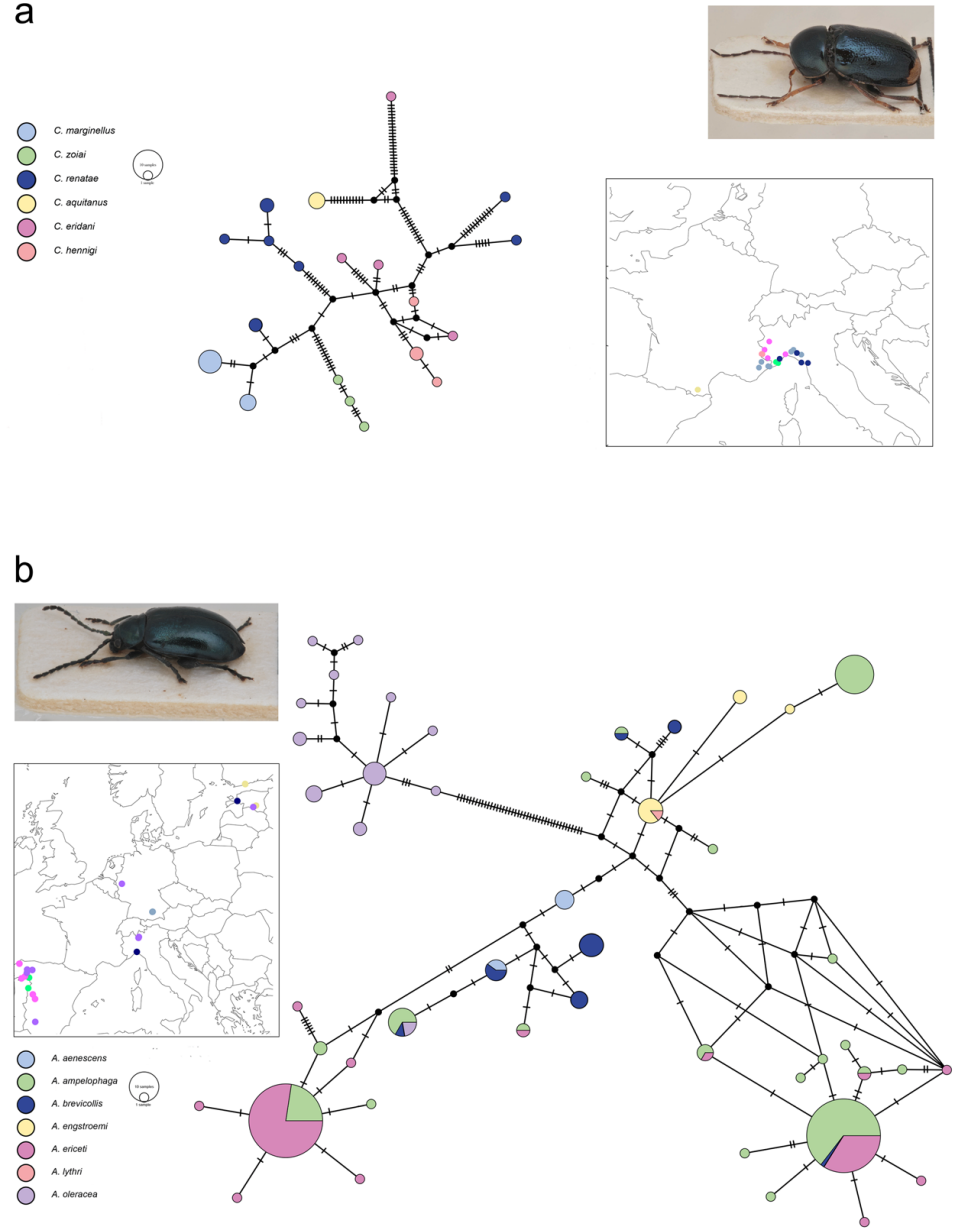
Minimum-spanning haplotype networks of COI sequences. (**a**) *Cryptocephalus marginellus* superspecies. (**b**) *Altica oleracea* species complex. For each group is reported an image of the representative species (*C*. *marginellus* and *A*. *oleracea*, respectively) and a map reporting collecting sites of the specimens included in this study. Diameter of the circle is proportional to haplotypes abundance.

The distributions of intraspecific and interspecific pairwise nucleotide distances overlap, thus resulting in the absence of a clear barcode gap in both family-level datasets (Fig. S1). The mean intraspecific nucleotide distance, estimated with the K2P nucleotide substitution model, resulted in 2% [0–20.6] for dataset *DS1* and 1.6% [0–27.6] for *DS2* (Figs 2 and S1). The exceptionally high maximum value of the intraspecific nucleotide distance in *DS1* of 20.6% is the result of the comparisons between sequences of two *Lachnaia tristigma* (Lacordaire, 1848) populations, both collected in France in the Alpes-de-Haute-Provence department. The interspecific nucleotide distance resulted in 25.1% [0–37.1%] in the case of *DS1* and of 24% [0–43.2%] in the case of *DS2* (Fig. S1). Noteworthy, 0 or close to 0 values of nucleotide distance were recovered between specimens belonging to different species (Fig. S1); among others, exemplar cases are represented by: *Cryptocephalus violaceus* Laicharting, 1781 - *Cryptocephalus duplicatus* Suffrian, 1845; *Lachnaia italica* Weise, 1881 - *Lachnaia tristigma*; members of *Cryptocephalus marginellus* Olivier, 1791 species complex, and of the *Cryptocephalus hypochaeridis* (Linnaeus 1758) species complex. In detail, specimens of *C*. *duplicatus* collected in Turkey and of *C*. *violaceus* collected in Greece possessed the same COI haplotype; within the *Cryptocephalus marginellus* complex, notable is the case of *Cryptocephalus renatae* Sassi, 2001 collected in Savona province having only ~0.6% of nucleotide distance from *C*. *marginellus* collected in a geographically close locality (Nice, FR) and of the *Cryptocephalus eridani* Sassi, 2001 having ~0.4% from *Cryptocephalus hennigi* Sassi, 2011, both collected in Cuneo province. The COI haplotype network of *C*. *marginellus* complex (Fig. 3a) confirms the previous results and shows that the currently known species in the group are not well distinguished as species clusters; the only exceptions are represented by *Cryptocephalus zoiai* Sassi, 2001 and *Cryptocephalus aquitanus* Sassi, 2001, unambiguously separated from the other species (Fig. 3a). In addition, within the subfamily of Alticinae some specimens belonging to the following eight out of 14 *Altica* morphospecies present in the *DS2* showed the same or highly similar COI haplotype (range of nucleotide intraspecific distances: 0–12.6% and of nucleotide interspecific distances: 0–13.8%): *Altica aenescens* (Weise, 1888), *Altica ampelophaga* Guérin-Meneville, 1858, *Altica ericeti* (Allard, 1859), *Altica brevicollis* Foudras, 1860, *Altica engstroemi* (Sahlberg, 1894), *Altica lythri* Aubé, 1843, *Altica longicollis* (Allard, 1860) and *Altica oleracea* (Linnaéus, 1758) (Fig. 3b).

### Optimal threshold and barcode efficiency

The optimal threshold that minimises the number of false positive and false negative identifications resulted in 2.6% of distance for *DS1*, with an associated cumulative error of 97 sequences out of 889 (10.9%, FP = 38, FN = 59); for *DS2* it resulted in a value of 1% of nucleotide distance, with a cumulative error of 816 sequences out of 7237, 11.3%, FP = 209, FN = 607). The sum of the cumulative errors obtained from optimal threshold analyses performed on the subfamily level datasets obtained from *DS1* resulted in 85 sequences (9.6%), and in 748 sequences (10.3%) in the case of datasets from *DS2*. (Table 1). These error values are significantly different from the cumulative errors obtained for the total datasets, i.e., *DS1*: t = −13.7, p-value < 0.001; *DS2*: t = −17.6, p-value < 0.001 (Table 1). The highest error values, related with subfamily datasets, were observed for Cryptocephalinae obtained from *DS1* (66 sequences out of 416, 15.8%, threshold 1%, Fig. 2) and for Alticinae from *DS2* (413 out of 2690 sequences, 15.3%, threshold 0.9%, Fig. 2). By contrast, the lowest error of only one sequence was obtained for both datasets of Cassidinae, with 53 sequences in *DS1* and 168 sequences in *DS2*; the associated OTs were higher than those observed for the other subfamilies, 4.6% for *DS1* and 5.9% for *DS2* (Fig. 2).

**Table 1:** Cumulative error values related to optimal thresholds of *DS1* and *DS2* datasets at family and subfamilies level.

Dataset IDs	Chr.	Alt.	Cas.	Chrys.	Cri.	Cry.	Don.	Eum.	Gal.	Ors.	His.	t test	p-value
*DS1*	97	10	1	3	1	66	2	0	1	0	0	−13.7	<0.001
*DS2*	816	413	1	40	16	192	12	0	73	0	0	−17.6	<0.001

Note. Abbreviations. Chr.: Chrysomelidae; Alt.: Alticinae; Cas.: Cassidinae; Chrys.: Chrysomelinae; Cri.: Criocerinae; Cry.: Cryptocephalinae; Don.: Donacinae; Eum.: Eumolpinae; Gal.: Galerucinae; Ors.: Orsodacninae; His.: Hispinae.

The barcode efficiency of *DS1*, evaluated through the best close match analysis gave an OT of 2.6%, resulting in 93% of correct identification (828 out of 889); 58 species, consisting of a single COI sequence, were considered as correctly identified since no match with other heterospecific sequences occurred. Of the 61 sequences that revealed identification errors, 14 were classified as incorrect identifications. These sequences belong to taxa between which very low interspecific nucleotide distances were observed (e.g., *C*. *hennigi* - *C*. *eridani*; *A*. *brevicollis* - *A*. *lythri*; *L*. *italica* - *L*. *tristigma*); in addition to these, they include also sequences from *Longitarsus apicalis* (Beck, 1817), showing the best match with *Longitarsus aeneicollis* (Faldermann, 1837) (pairwise nucleotide distance of 0.2%), and one specimen of *Oulema melanopus* (Linnaeus, 1758) that matched with *Oulema duftschmidi* (Redtenbacher, 1874) (1.2% of pairwise nucleotide distance). A total of 39 sequences (34 morphospecies) resulted in no match with conspecifics because of a pairwise nucleotide distance higher than the adopted OT. Among these cases, a sequence of *Cassida denticollis* Suffrian, 1844 showed about 15% of nucleotide divergence from other sequences assigned to the same species. The eight ambiguous identifications involve the sister species *C*. *violaceus - C*. *duplicatus* and *L*. *tristigma*.

The same analysis performed on *DS2* highlighted the presence of 94.1% correct identifications (6,811 sequences out of 7,237), 52 incorrect, 164 ambiguous and 210 missing identifications (Table S2), with an OT of 1%. Among incorrect and ambiguous identifications, beyond the *DS1* cases mentioned above, only one match involved at least one sequence from *DS1* (i.e., *Psylliodes brisouti* Bedel, 1898 specimen code MS0000647 with *Psylliodes instabilis* Foudras, 1860 accession number KM445439). Incorrect and ambiguous identifications were observed also among the retrieved sequences: e.g. one sequence of *L*. *tristigma* and one of *Lachnaia gallaeca* Baselga & Ruiz-García, 2007 (nucleotide distance 0.2%); *Plateumaris sericea* (Linnaeus, 1761) and *Plateumaris discolor* (Panzer, 1795) sequences and, *Galerucella pusilla* (Duftschmid, 1825) and *Galerucella calmariensis* (Linnaeus, 1767). As regard the missing identifications, sequences assigned to 11 species of *Cassida*, 27 of *Cryptocephalus* and 6 of *Smaragdina* genera did not match those of conspecifics because of intraspecific genetic distances higher than the OT.

## Discussion

### Identification efficiency

The results achieved by the performed analyses confirmed the usefulness of the DNA barcoding approach as a tool for the molecular identification of Chrysomelidae. The obtained identification efficiencies are comparable for both datasets; our dataset (*N*_*DS1*_ = 889 sequences) showed 93% of correct identifications, while 94% of correct identifications was obtained for *DS2* (i.e., the available COI sequences +*DS1*; *N*_*DS2*_ = 7,237 sequences), which cover the ~24% of the Euro-Mediterranean species. The barcoding efficiency recovered in the present study is similar to those achieved in other studies dealing with beetles, as example 89% in the case of *Bembidion* species^36^, approximately 92% in the case of the Central European Coleoptera (39% of the fauna)^19^ and 100% in the case of *Crioceris* species^37^. In any case, in these studies different approaches were adopted to estimate the barcoding efficiency, thus a direct comparison could not be performed.

Incorrect, ambiguous and missing identifications observed in our study are possibly related with the inability of DNA barcoding in identifying taxa in the presence of: (*i*) superspecies (two or more close related species with allopatric distribution that can occasionally hybridise^38^) and cryptic species complexes^39,40^; (*ii*) cases of hybridisation or introgression; (*iii*) incomplete lineage sorting; and (*iv*) bacterial endosymbionts changing pathways of mtDNA inheritance^41,42^. In these cases, the lack of a clear barcode gap between intraspecific and interspecific nucleotide distances vanish the possibility to identify species^32,43^. The phenomenon is evident also in the analysed datasets, where a clear barcode gap cannot be found (Fig. S1).

Interestingly, the estimated optimal threshold of *DS2* was lower than that of *DS1*, 1% and 2.6% respectively. These results could be related to the different haplotype diversity and to the different taxonomic composition of the two datasets. The mean number of haplotypes per species of the two datasets is 6.7 (on average 13.4 sequences per species) and 2.5 (on average 3.4 sequences per species) in the case of *DS2* and *DS1*, respectively; thus, *DS1* possesses fewer sequences per species but a higher number of haplotype per species (approximately one haplotype per sequence) than *DS2*. The differences between the two datasets might be related to the sampling strategies adopted in C-Bar project, where attempts have been made to maximise the number of conspecifics from different localities, rather than to process numerous specimens of the same species from the same locality.

Threshold optimisation analyses showed also a significant decrease of the cumulative error when OTs were estimated at the subfamily level in comparison to when they were estimated at the family level (Table 1). Phylogenetically closely related species are supposed to have similar rates of nucleotide substitution due to shared morphological, biological and ecological traits (*e*.*g*., number of generation per year, tendency to isolation of the populations due to the habitat structure or to the dispersal ability of the species^44^, and for this reason should be easier to define a reliable threshold between intraspecific and interspecific divergence. We can hypothesise that not all Chrysomelidae share the same rate in nucleotide substitutions, since different subfamilies are characterised by different morphological, ecological and physiological adaptation, as the Maulik’s organ that confers jumping capabilities to Alticinae^45,46^, the limited dispersal capabilities of Chrysomelinae and Cryptocephalinae^47^ or the presence of bacterial endosymbiont that, in the case of Donacinae, allows the larvae to survive in anoxic conditions under water^48^. Moreover, the different OTs achieved for Chrysomelidae subfamilies underline that the use of a unique threshold for the entire family decreases the identification efficiency of DNA barcoding (Table 1). Beyond classical barcoding studies, the implementation of group specific thresholds, leading to a more accurate taxonomic identification, should be also evaluated for OTUs clustering in metabarcoding analyses instead of the employment of fixed thresholds (as in the case of^49,50^).

Concerning the cumulative error, the highest value was obtained for Cryptocephalinae subfamily (*DS1*). This dataset, accounting for 46.8% of *DS1* sequences, includes different species complexes (e.g., *Cryptocephalus marginellus* superspecies and *Cryptocephalus hypochoeridis* complex). The presence of species complexes increases the overlap between intra and interspecific distances and consequently the cumulative error at the optimal threshold. In the case of *DS2*, Alticinae resulted the subfamily with the highest error associated to the OT of 0.9%. This finding could be associated to a high proportion of sequences belonging to the genus *Altica* in this dataset (229 out of 2,690), a taxon for which inconsistences between molecular and morphological signals were already found^51^.

### Molecular identification of closely related species

Barcode sequences of closely related species within the groups *Cryptocephalus hypochaeridis*^52,53^ and *Oulema melanopus*^54,55^ were here analysed; as expected, low values of nucleotide interspecific distances within groups were observed. Moreover, our study highlighted other interesting cases of sequences belonging to morphologically similar species groups not properly identified by best close match analyses. *Cryptocephalus marginellus* superspecies, including six species that differ in their distributions and in the shape of the median lobe of aedeagus^56,57^ represents one of these cases. These species are present in Spain (*C*. *aquitanus*), France (*C*. *aquitanus*, *C*. *marginellus*, *C*. *eridani* and *C*. *zoiai*), Italy (*C*. *eridani*, *C*. *marginellus*, *C*. *renatae*, *C*. *hennigi* and *C*. *zoiai*) and Switzerland (*C*. *eridani*), and their distributions partially overlap in some areas. The close relationships among these species highlighted by morphological features were here confirmed by the COI variability and by the structure of the haplotype network (Fig. 3a); however, no shared haplotypes between species were observed. Well-separated clusters were recovered for *C*. *aquitanus* and *C*. *zoiai* that, in addition to *C*. *marginellus*, resulted the only monophyletic taxon within this group (Fig. 3a). The analysis of pairwise nucleotide distances showed low values between different species, the lowest one between specimens collected in the area where the range of the species overlap (e.g., *C*. *eridani* - *C*. *hennigi*). Incomplete lineage sorting could be considered an explanation for these results, even if introgression between species with overlapping distribution has to be taken into account. A further interesting result concerns *C*. *violaceus* and *C*. *duplicatus*, two morphologically very similar species distinguishable only on the basis of the shape of the median lobe of the aedeagus. *C*. *violaceus* is present in central and southern Europe while *C*. *duplicatus* in the southern east of Europe and the Middle east. No nucleotide differences were observed between the COI sequences of *C*. *violaceus* collected in Greece and *C*. *duplicatus* collected in Turkey. Since the distribution of the species overlaps in Greece, we can hypothesise recent events of introgression. This phenomenon is known to occur when, after an allopatric speciation, two sister species come in contact and establish an area of secondary sympatry; due to the lack of reproductive isolation they have the possibility to hybridise with the result of a stable integration of genetic material from one species into the other one^58,59^.

Shared haplotypes were observed among the following *Altica* species: *A*. *ericeti - A*. *ampelophaga; A*. *ampelophaga - A*. *oleracea - A*. *brevicollis; A*. *brevicollis - A*. *aenescens; A*. *ericeti - A*. *ampelophaga - A*. *brevicollis; A*. *ampelophaga - A*. *brevicollis; A*. *lythri - A*. *engstroemi*. Identification of many species belonging to *Altica*, included those above mentioned, is not easy adopting morphological criterion; it is mainly based on the observation of adult male genitalia, which in some cases is not totally informative because of the presence of intraspecific morphological variation^60^. In addition, adult females are often indeterminable^61^. This difficulty in species identification is also mirrored at the molecular basis, where the species of this group are unidentifiable using COI (Fig. 3b) as well as by using other mtDNA markers^51^. Morphological and COI nucleotide similarity suggests the possible need of a taxonomic revision of the *Altica* species mentioned above. The obtained results, *viz* the low interspecific nucleotide divergence and the presence of shared haplotypes, is congruent with a scenario of incomplete lineage sorting due to the recent origin of the group of species and hybridization. A further possibility, supported by the presence of different strains of the maternally inherited endosymbiont *Wolbachia* within and between *Altica* species, consists of a rapid spread within populations of ancestral or introgressed haplotypes, caused by the cytoplasmic incompatibility induced by *Wolbachia*^51^. In this last scenario, *Wolbachia* might have played a crucial role in mating isolation and thus in the speciation process, as suggested for other groups of close related taxa (e.g.^62–64^). Further studies, using genomic approaches, are required to disentangle among the reported possibilities.

## Conclusion

This study provides COI sequences of 261 Chrysomelidae species (~12% of the Euro-Mediterranean Fauna; 889 barcodes) collected in the Euro-Mediterranean area (52 species new to on-line repositories) and confirms the usefulness and efficiency of DNA barcoding for the identification of these beetles. Cases of barcoding failure in identifying members of the family were observed especially for closely related species, and some of them are reported for the first time in this study. The comparisons among optimal thresholds estimated at different taxonomic levels, *viz* family and subfamily, have underlined the importance of using taxon-specific thresholds to increase the efficacy of molecular identification.

## Electronic supplementary material

Figure S1

Supplementary Tables 1-5

## Data Availability

All the COI sequences and the metadata associated with the organisms processed in this study are available in Bold Systems, European Nucleotide Archive and Supplementary Tables S1 and S3. Voucher specimens are deposited into M.M. private collection.
