# Supplementary material for: Barcoding of Chrysomelidae of Euro-Mediterranean area: efficiency and problematic species
Source: Sci Rep. 2018 Sep 7;8:13398. doi: 10.1038/s41598-018-31545-9 (PMC6128942; doi:10.1038/s41598-018-31545-9)
Supplement: Supplementary file 1 — Figure S1 [file 41598_2018_31545_MOESM1_ESM.docx]

***Title page***

Barcoding of Chrysomelidae of Euro-Mediterranean area: efficiency and problematic species

***Authors***

Giulia Magoga^1^, Didem Coral Sahin^2^, Diego Fontaneto^3^, Matteo Montagna^1*^

*corresponding author: [matteo.montagna@unimi.it](mailto:matteo.montagna@unimi.it)

***Author Affiliation***

^1^Dipartimento di Scienze Agrarie e Ambientali - Università degli Studi di Milano, Via Celoria 2, 20133 Milano, Italy.

^2^Directorate of Plant Protection Central Research Institute, Yenimahalle, Ankara, Turkey.

^3^Consiglio Nazionale delle Ricerche-Istituto per lo Studio degli Ecosistemi, Largo Tonolli 50, 28922 Verbania, Italy.


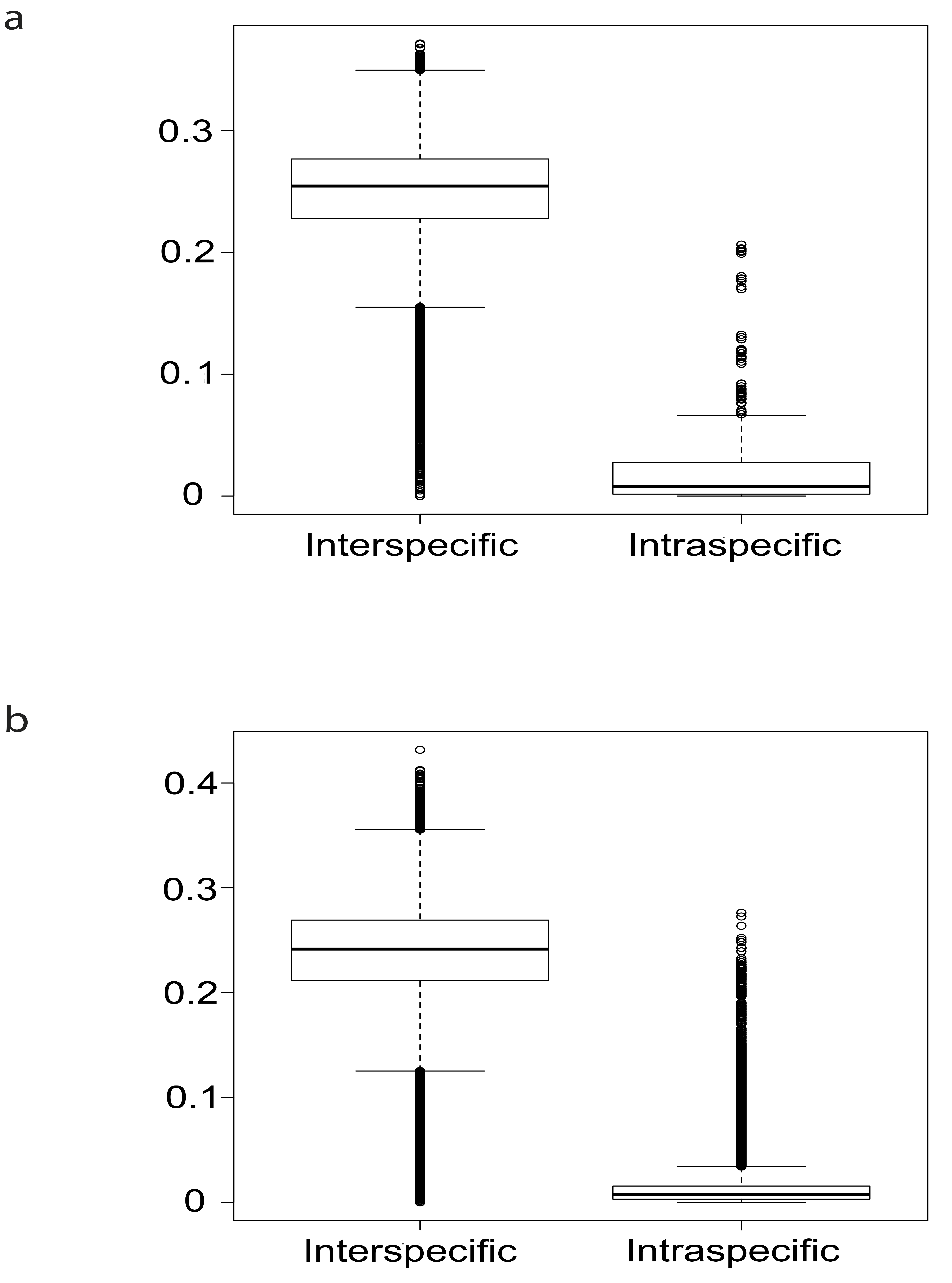


Supplementary Figure S1. Boxplots of K2P inter-intraspecific pairwise nucleotide distances of *DS1* (a) and *DS2* (b).
